# Clinicopathological features and genomic profiles of a group of secretory breast carcinomas in which progressive cases have more complex genomic features

**DOI:** 10.1186/s13000-022-01284-7

**Published:** 2022-12-31

**Authors:** Ting Lei, Yuyan Yang, Yongqiang Shi, Xu Deng, Yan Peng, Hui Wang, Tongbing Chen

**Affiliations:** 1 Department of Pathology, The Third Affiliated Hospital of Soochow University, Changzhou, Jiangsu 213003 P.R. China; 2 Department of Pathology, The Second Hospital of Tianjin Medical University, Tianjin, 300211 P.R. China

**Keywords:** Secretory breast carcinoma, *ETV6-NTRK3*, *TERT*, Tumor progression

## Abstract

**Background:**

Secretory breast carcinoma (SBC) is a rare malignant breast neoplasm with distinct histological features, including solid, microcystic, tubular, and rarely papillary structures, traditionally characterized by a t (12;15) (p13:q25) translocation, which usually leads to *ETV6-NTRK3* fusion, suggesting an early event in tumorigenesis. Due to the rarity of this disease, very few genome sequencing studies have been performed on a series of cases, especially progressive cases.

**Methods:**

Seven lesions from 5 patients diagnosed at the Third Affiliated Hospital of Soochow University from 2007 to 2021 were included. Clinicopathological features and prognosis/survival data were collected. Next-generation DNA sequencing was performed on six of the seven lesions.

**Results:**

In total, 3/7 (42.9%) lesions demonstrated estrogen receptor (ER) expression, including weak, moderate to strong staining, and no lesion demonstrated progesterone receptor (PR) expression. There were no cases of human epidermal growth factor (HER2) overexpression, and the Ki-67 index was low. S-100 and pan-TRK protein were diffusely positively expressed in all cases. All lesions were characterized by a t(12;15) (p13:q25) translocation, leading to *ETV6-NTRK3* fusion confirmed by fluorescence in situ hybridization (FISH). The sequencing results showed that *ETV6-NTRK3* fusion was the main driver of early tumorigenesis, while SBC with invasive biological behavior had more complex genomic variation in which *TERT* promoter mutation was detected.

**Conclusions:**

Immunohistochemical staining of a biomarker panel, including ER, PR, HER2, Ki-67, S-100 and pan-TRK, can be used as an auxiliary diagnostic tool, and FISH detection can be used as a diagnostic tool. *ETV6-NTRK3* gene fusion involving multiple sites may drive tumorigenesis, while mutations in the *TERT* promoter region may be a factor driving tumor progression.

## Introduction

Secretory breast carcinoma (SBC) is a rare breast cancer accounting for <0.05% of all invasive mammary carcinomas and was initially designated “juvenile carcinoma” by McDiwitt and Stewart in 1966 [1]. Although first described in children, SBCs affect more adults than children, with a median age of 53 years (ranging from 3 to 91 years) [1–3].

SBCs are traditionally characterized by a t(12;15) (p13:q25) translocation, leading to *ETV6-NTRK3* fusion that presents both in the in situ and invasive components, suggesting that it is an early event in tumorigenesis [4–9]. *ETV6-NTRK3*, originally identified in congenital fibrosarcoma and cellular mesoblastic nephroma, encodes a constitutively activated chimeric tyrosine kinase that drives cellular transformation and oncogenesis through the RAS-MAPK and PI3K pathways [5–7, 10, 11]. One recent study found that in addition to *ETV6-NTRK3* fusion, fusion of *NTRK1* and other genes can also drive SBCs [12]. Transfer of *ETV6-NTRK3* retrovirus into mouse mammary epithelial cells produced tumor-prone transformed cells in nude mice, but it is not clear whether the gene can drive tumorigenesis on its own or if additional modifications are required [6].

SBCs generally have an indolent clinical course, even in patients with axillary node metastasis [13]. In one population-based study, the 5- and 10-year cancer-specific survival rates were 94% and 91%, respectively [14]. Another study based on data from the National Cancer Database (NCDB) demonstrated that the overall survival of SBC patients was better than that of invasive ductal carcinoma (IDC) patients [15]. Given this indolent behavior, tumors are usually managed conservatively and routinely removed by surgery; there are nonspecific guidelines for radiotherapy and chemotherapy, with varying treatment regimens in the literature [14–16]. As more studies are conducted, it seems necessary to predict outcomes beyond the commonly used 5 or 10 years for diseases such as IDC, but longer for SBC. Most recurrence and metastasis events occur after 5 or even 10 years [17–19]. However, cases with rapid progression and a poor prognosis also exist [20, 21]. Cases with different biological behaviors show no obvious differences in histology, immunophenotype, etc. in studies thus far. The advent of next-generation sequencing (NGS) technology has provided an opportunity to further understand the genomic characteristics of such tumors, possibly providing clues to different behaviors. Due to the rarity of this disease, very few genome sequencing studies have been performed on a large number of cases [5, 12], and most sequencing studies are usually case reports involving one or two subjects [2, 11, 18, 22].

To gain greater insights into the biological characteristics of SCBs, especially those that showed disease progression. We described the morphology and immunophenotype of seven lesions in five SBC patients and used capture-based next-generation sequencing of 425 cancer-related genes to more comprehensively characterize the genomics of more SBCs based on previous reports.

## Materials and methods

### Patients

The study was approved by the Institutional Review Board of the Third Affiliated Hospital of Soochow University. Five SBC patients were identified in the pathology department archives of the Third Affiliated Hospital of Soochow University, spanning from 2007 to 2021. All specimens were fixed in 10% neutral buffered formalin and embedded in paraffin. Clinical information was obtained from the electronic medical records. All of the sections and pathological features were reviewed by two pathologists (T.L., and TB.C.) in the Third Affiliated Hospital of Soochow University. Survival outcomes were evaluated through telephone follow-ups. The disease-free survival (DFS) interval was calculated from the diagnosis of the disease until the first instance of disease progression.

### Immunohistochemistry (IHC)

IHC analysis was performed for estrogen receptor (ER) (Ventana, Tucson, Arizona, USA), progesterone receptor (PR) (Ventana, Tucson, Arizona, USA), human epidermal growth factor (HER2/neu) (Ventana, Tucson, Arizona, USA), Ki-67 (Ventana, Tucson, Arizona, USA), S-100 (1:100, MXB Biotechnology, Fujian, China), and pan-TRK (1:100, ZSGB- BIO, Beijing, China). After dewaxing and hydration, 4 µm formalin-fixed paraffin-embedded (FFPE) tissue sections were treated with an immunohistochemical automatic staining machine according to the scheme provided by the Benchmark XT system. For ER, PR, and HER2, positive staining was defined according to American Society of Clinical Oncology/College of American Pathologists (ASCO/CAP) guidelines. For all other markers, positive expression was defined as cytoplasmic (S-100) or nuclear (pan-TRK) staining.

### Fluorescence in situ hybridization (FISH)

For *ETV6* fluorescence in situ hybridization, 4-μm (FFPE) tumor sections were baked at 60 °C for 1 h, rinsed in 100% ethanol, and then pretreated with 0.2 N HCl (20 min, room temperature), followed by 1 M NaSCN (30 min, 80 °C) before protease digestion with pepsin (2.5 mg/ml pepsin for 27 min at 37 °C). The slides were then fixed in 10% phosphate-buffered saline-buffered formalin, rinsed, dehydrated in an ethanol series, and air dried. The slides were hybridized overnight at 37 °C with a commercial *ETV6* break-apart probe (Vysis *ETV6* Break Apart FISH Probe Kit, Abbott) according to the manufacturer’s instructions. The slides were washed to remove unbound probes and counterstained with 4,6 diamidino-2-phenylindole. Enumeration of break-apart signals was conducted using a Zeiss fluorescence microscope.

### DNA isolation

DNA was extracted from FFPE representative 5-μm tumor tissues using a QIAamp DNA FFPE Tissue Kit (Qiagen, Cat No. 56404) according to the manufacturer’s instructions. The quality of DNA was verified by migration on agarose gel, and the concentration of DNA was quantified by spectrophotometer (Nanodrop 2000, Thermo).

### Capture-based next-generation DNA sequencing

Sequencing was performed at a commercial laboratory using an assay that targets the coding regions of 425 cancer-related genes. For the commercial next-generation sequencing (NGS) panel, the mean (*n =* 41) mapped reads per sample was 463127 (range 111595-646854), the mean depth per base was 3811 (range 983-6145) and the mean reads on the target sequence was 94.57% (range 71.73%-98.71%). Original image data were transferred by base calling analysis into raw sequence data. Single nucleotide variants (SNVs) and short insertions/deletions (indels) were identified by VarScan2 with the minimum variant allele frequency threshold set at 0.01 and a *p* value threshold for calling variants set at 0.05 to generate Variant Call Format files. All SNVs/indels were annotated with Annotate Variation (ANNOVAR), and each SNV/indel was manually checked on the Integrative Genomics Viewer. Copy number variation analysis was performed using an inhouse developed pipeline. A fold change threshold of 1.6 and 0.6 in DNA copy number was set as the cutoff for amplification and deletion, respectively.

### Statistical analysis

Patient clinicopathological characteristics and outcomes are reported as number (%), mean ± SD, or median (95% confidence interval [CI]), as appropriate.

## Results

### Clinical characteristics and outcomes

The clinicopathologic features, treatment, and follow-up data of SCB patients are summarized in Table 1. All five patients were female, with a median age of 39 years and a mean age of 34.8 ± 3.4 years (range from 33 to 42 years) at first onset. Tumor size ranged from 0.9 to 7.0 cm, with a median size of 2.0 cm and a mean size of 2.7 ± 2.21 cm. Of the five primary cases, three were located in the left mammary gland, and two were located in the right mammary gland, one of which was associated with axillary lymph node metastasis.

**Table 1:** Clinicopathologic features of breast secretory carcinomas

Patient	Sex	Age	Position	Associated in situ carcinoma	Tumor size (cm)	Lymph node metastasis	Pathological stage	ER	PR	HER2	Ki-67	S-100	Pan-TRK	Treatment	Follow-up (month)
1	Female	39	Left	No	1.0	0	T1N0M0	Weak positive	negative	negative	5%	positive	positive	mastectomy, LND, CT	140m contralateral SBC
	Female	51	Right	No	1.1	0	T1N0M0	Weak positive	negative	negative	5%	positive	positive	mastectomy, LND, CT	30m ANED
2	Female	42	Right	Yes	4.0	2	T2N1M0	negative	negative	negative	30%	positive	positive	mastectomy, LND, CT	84m recurrence and distant metastasis
	Female	49	Chest wall	No	2.5	NA	-	negative	negative	negative	35%	positive	positive	L,CT,RT,	5m ANED
3	Female	41	Right	No	2.0	0	T1N0M0	moderate to strong positive	negative	negative	15%	positive	positive	L, LND, CT,RT, HT	18m ANED
4	Female	33	Left	No	7.0	0	T2N0M0	negative	negative	negative	10%	positive	positive	L, LND	10m ANED
5	Female	39	Left	No	0.9	0	T1N0M0	negative	negative	negative	20%	positive	positive	mastectomy, LND,	8m ANED

*NA* Not available, *CT* Chemotherapy, *RT* Radiotherapy, *HT* Hormonotherapy, *ANED* Alive with no evidence of disease, *ER* Estrogen receptor, *PR* Progestogen receptor, *HER2* Human epidermal growth factor 2, *LND* Lymph node dissection, *L* Lumpectomy, *m* Month, *T* Tumor size, *N* Regional lymph node, *M* Distant metastasis

The follow-up time ranged from 8 to 170 months. Patient 1 developed contralateral SBC 140 months after the occurrence of left SBC, and Patient 2 developed chest wall recurrence and distant metastasis 84 months after diagnosis. Patient 5 had concurrent primary breast tumors, including SBC and invasive ductal carcinoma (IDC), with no signs of disease progression during an 8-month follow-up. Patient 3 and Patient 4 both had a single lesion, and the follow-up time was short without disease progression. All five (5/5, 100%) patients underwent mastectomy or lumpectomy plus axillary lymph node dissection, in which three of five (3/5, 60%) patients received chemotherapy for the first onset. The patient with moderate to strong ER expression also received radiotherapy and hormonal therapy in addition to chemotherapy (Table 1).

### Pathological features and immunohistochemical profiles

**Fig. 1:**
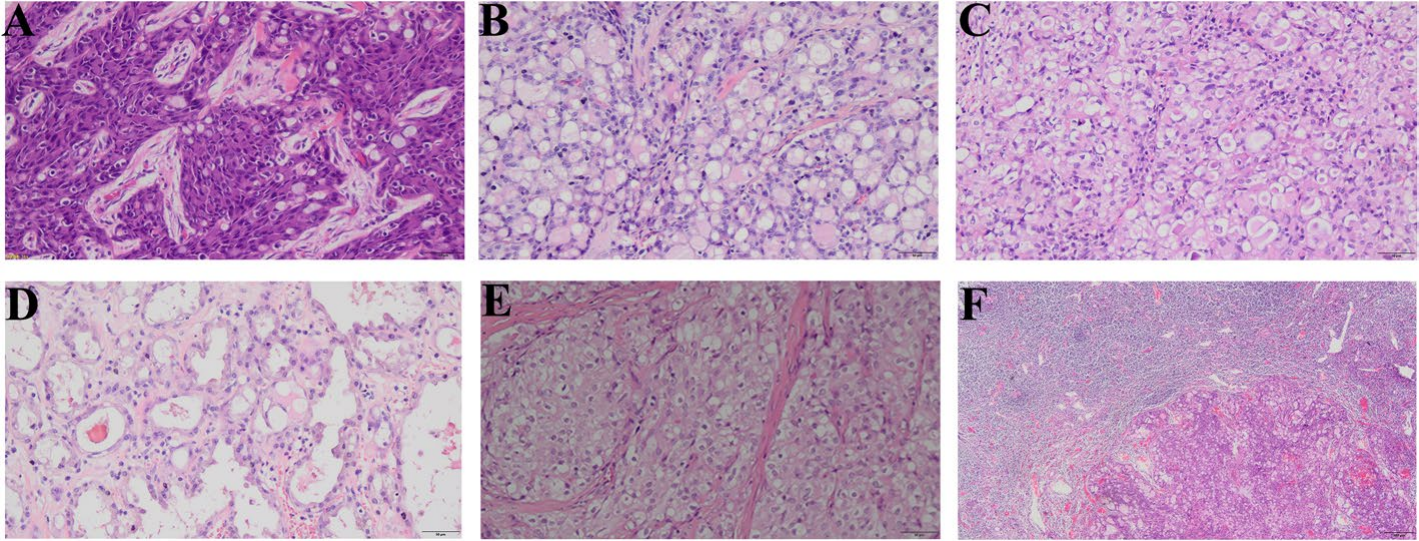
SBC (hematoxylin and eosin staining). **A** Solid area separated by a fibrous septum with intracellular mucinous vacuoles and eosinophilic secretory vacuoles. **B**, **C** Solid and microcystic structure with intracellular and extracellular vacuoles. **D** Tubular area with intracellular and extracellular eosinophilic vacuoles. **E** Solid structure separated by fibrous septum with extracellular eosinophilic secretion. **F** Metastatic SBC components in axillary lymph nodes in Patient 2

All the cases in our study showed a typical SBC morphology composed of polygonal cells with eosinophilic or vacuolated cytoplasm. Round or oval nuclei were arranged in a microcystic, cystic or solid growth pattern in which intracytoplasmic and extracellular amphophilic or eosinophilic secretion existed (Fig. 1). Only one recurrent case showed extensive fibrotic segmented cords and glandular tubular structures. The histological features of every case are shown in Table 2.

**Table 2:** The histological features of SBCs

Patient	Position	Structure	Cell morphology	Boundary
1	Left	microcystic, solid	cytoplasmic vacuolization, eosinophilic secretion	circumscribed borders, focally infiltrate surrounding fat tissue
1	Right	Solid, microcystic	cytoplasmic vacuolization, extracellular eosinophilic secretion	circumscribed borders
2	Right	Tubular, Solid, microcystic, cystic	intracellular and extracellular eosinophilic secretion. cytoplasmic vacuolization	infiltrate surrouding tissue
2	Chest wall	Tubular, Solid, microcystic, cystic	intracellular and extracellular eosinophilic secretion. cytoplasmic vacuolization	infiltrate surrounding fat tissue and striated muscle
3	Right	microcystic, cystic	intracellular and extracellular eosinophilic secretion. cytoplasmic vacuolization	infiltrate surrounding fat tissue
4	Left	Solid, microcystic, cystic	intracellular and extracellular eosinophilic secretion. cytoplasmic vacuolization	circumscribed borders, focally infiltrate surrounding fat tissue
5	Left	microcystic	intracellular and extracellular eosinophilic secretion. cytoplasmic vacuolization	infiltrate surrounding fat tissue

**Fig. 2:**
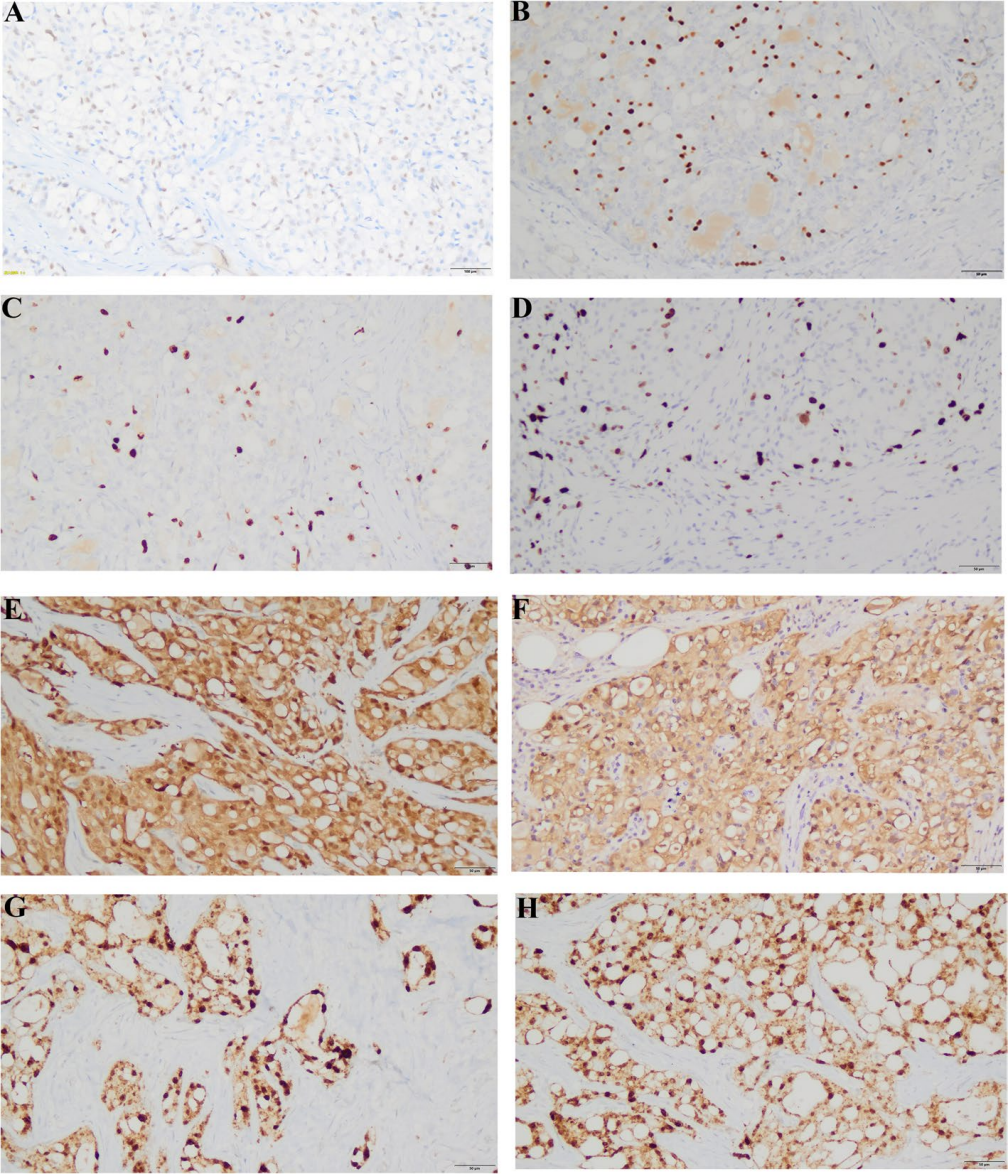
Immunohistochemistry staining of SBC. **A** Weak positive ER staining. **B** Moderate to strong positive ER staining. **C** Relatively low Ki-67 index/immunoreactivity. **D** Relatively high Ki-67 index/immunoreactivity. **E**, **F** Diffuse and strong S-100 immunoreactivity. **G**, **H** Strong positive nuclear staining for Pan-TRK in all cases

In all seven lesions, three lesions (3/7, 42.9%) demonstrated ER expression, and no lesion demonstrated PR expression. There were no HER2 overexpression cases. The Ki-67 index ranged from 5% to 35% and was relatively higher in the primary and recurrent lesions of patients with recurrence and distant metastasis. S-100 was diffusely positive in all the cases, and NTRK was diffusely and strongly positively expressed in all nuclei in all cases (Fig. 2).

### Genomics of SBCs

**Fig. 3:**
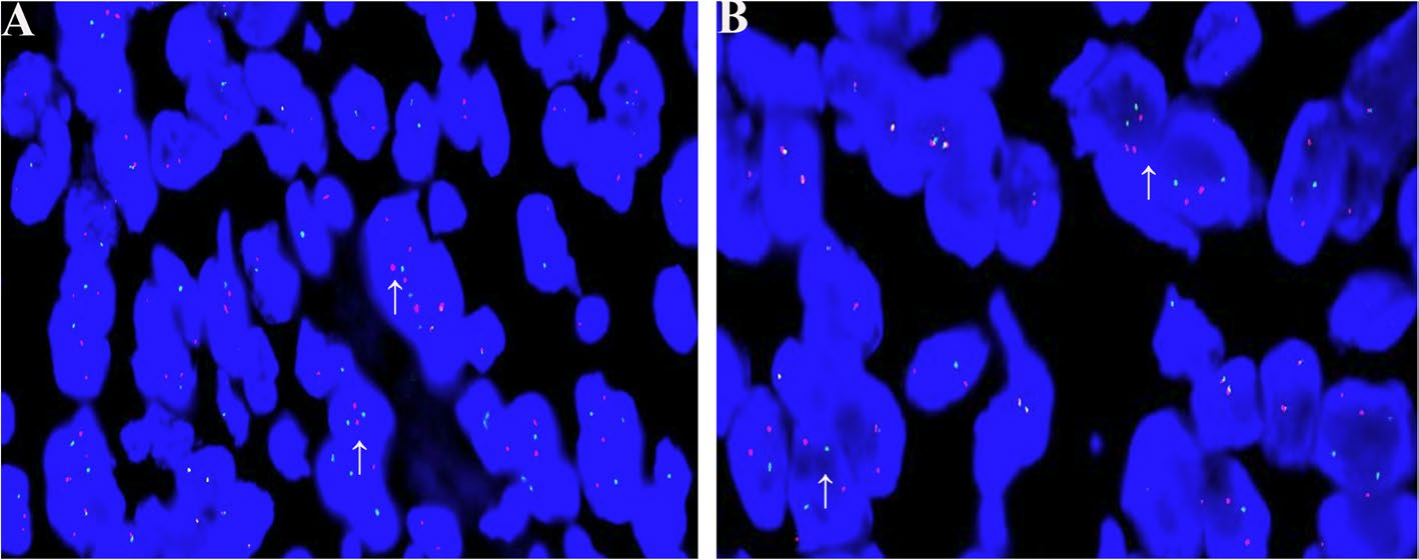
Fluorescence in situ hybridization analyses for *ETV6* breaks apart in SBC. FISH images showed *ETV6* break-apart signals in epithelial cells

All seven lesions were available for *ETV6* break-apart probe detection, in which all the lesions showed break-apart signals (Fig. 3).

**Fig. 4:**
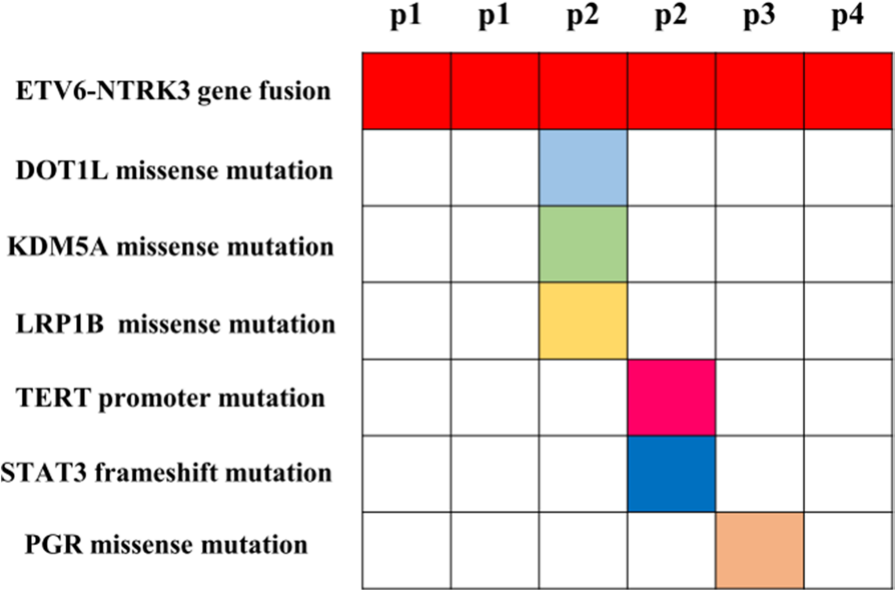
Summary of next-generation sequencing results for 6 lesions in 4 secretory carcinoma patients. All lesions demonstrated *ETV6–NTRK3* gene fusion by DNA sequencing. Patient 2 sequencing results showed *DOT1L*, *KDM5A*, *LRP1B* variants in primary lesions and *TERT* and *STAT3* mutation variants in recurrent lesions, except for the same *ETV6-NTRK3* gene fusion sites. Patient 3 had two *ETV6-NTRK3* gene fusion sites, and *PGR* gene variation was detected.

Six of the seven lesions were available for 425 cancer-related gene DNA sequencing detections. All six SBCs revealed *ETV6–NTRK3* gene fusions by DNA sequencing, showing a variety of fusion sites, including exon 5 of *ETV6* and exon 15 of *NTRK3,* which were the most frequent alterations; exon 6 of *ETV6* and exon 15 of *NTRK3*; and exon 6 of *ETV6* and exon 14 of *NTRK3*. Patient 1, who developed asynchronous SBCs in the bilateral breast; this patient showed different *ETV6–NTRK3* gene fusion sites in the two neoplastic tissues and exhibited no other alterations. In patient 2, except for the same *ETV6-NTRK3* gene fusion sites, *DOT1L*, *KDM5A*, and *LRP1B* were also existed in the primary lesion, and *TERT* and *STAT3* mutations were detected in recurrent lesions. Patient 3 had two *ETV6-NTRK3* gene fusion sites, exon 5 of *ETV6* and exon 15 of *NTRK3* and exon 6 of *ETV6* and exon 15 of *NTRK3*. *PGR* gene variation was also detected. Only the fusion of exon 5 of *ETV6* and exon 15 of *NTRK3* was detected in patient 4 (Fig. 4). All the lesions showed a low tumor mutation burden (TMB) ranging from 0 mut/Mb to 3.2 mut/Mb, and recurrent lesions had the highest TMB values.

## Discussion

SBCs are rare malignant neoplasms of the breast with distinct histological features, including solid, microcystic, tubular, and rarely papillary structures [1, 6, 18]. Recent research has revealed that in addition to *ETV6-NTRK3* fusion, fusion of *NRTK1* with other gene partner genes also drives tumorigenesis [12]. Although an increasing number of studies on SBC have been reported, gene sequencing studies of multiple cases, especially recurrent and metastatic cases, are still limited. In our study, clinicopathological features, treatment modalities, and prognostic information of 7 lesions in 5 patients with SBCs were summarized, and the genomic characteristics of these patients were analyzed using next-generation sequencing technology.

All our cases showed typical morphological features with round or oval nuclei arranged in microcystic or solid growth patterns and intracellular and extracellular eosinophilic or eosinophilic secretions. A panel of IHC markers, including ER, PR, HER2, Ki-67, S-100 and Pan-TRK [2, 23–25], may serve as a useful tool to aid the diagnosis of SCB. Initial studies have shown that SBC is usually triple negative or weakly positive for hormone receptors with S-100 positive expression [26–28]. With further research, we found that cases with moderate or even strong ER positivity also existed and that S-100 is expression is not specific to SBCs [4, 5, 27]. With the development of molecular detection technology, pan-TRK protein, which recognizes a conserved sequence near the C-terminus of TRK proteins, has been found to have certain sensitivity and specificity in *ETV6-NTRK* fusion tumors [23, 24, 29]. The literature shows that the unique morphology of the tumor combined with diffuse and at least focal strong nuclear staining can be used for the initial diagnosis in most cases [23, 24, 30]. FISH is still recommended for patients with atypical histomorphology or negative pan-TRK protein expression [23, 24]. Since alternations in both *NTRK3* and *NTRK1* drive tumorigenesis, it is important to use pan-TRK immunohistochemical staining in the initial screening and to use multiple probes for FISH detection. Our immunohistochemical results showed that pan-TRK protein was localized in the nucleus and diffuse and strongly stained in all cases, and *ETV6* break-apart probes were positive for FISH detection.

Further revealing the genomic characteristics of SBC can provide a better understanding of the biological behavior of this disease and provide clues for its prognosis and treatment. To comprehensively describe the gene expression profile of SBC, we summarize the molecular information of SCB detection by NGS in the literature in Table 3 as follows: Krings’ study showed that SBC is mainly driven by fusion of the *ETV6-NTRK3* gene, and the DNA breakpoints were mostly in *ETV6* intron 5 and *NTRK3* intron 14 [5]. In addition, fusion of exon 5 of *ETV6* and exon 15 of *NTRK3* was detected in two SBCs with distant metastasis, and additional gene variants were present in the two SBCs [18, 22]. Maund *et* al. detected additional fusion driver genes (*LMNA*-*NTRK1* and *TPM3*-*NTRK1*) and some other pathogenic genes, including *CCND1*, *TERT*, *EMSY*, and *CDH1 et al* [12]. *UGT1A1* variants other than *ETV6-NTRK3* were found in SBCs with lymph node metastases reported by Chen *et al* [2]. Compared with the above studies, our study presented more diverse *ETV6-NTRK3* gene fusion sites. Although only one case in this study had a mutation in the *TERT* promoter region, this mutation has been detected in previous studies and in recurrent lesions [12, 18, 22]. The diverse fusion sites of the *ETV6* and *NTRK3* genes included exon 5 of *ETV6* and exon 15 of *NTRK3*, exon 6 of *ETV6* and exon 15 of *NTRK3*, and exon 6 of *ETV6* and exon 14 of *NTRK3*. The duplication of *ETV6-NTRK3* reported in Krings’ Lambros’ and Chen’s study was not detected in our research [2, 5, 11]. It has been shown that the *ETV6–NTRK3* fusion gene encodes a chimeric tyrosine kinase that signals through Ras-mitogen-activated protein kinase and phosphatidylinositol-3 kinase pathways to transform cells of multiple lineages, regardless of the molecular type of SBC [5, 6].

**Table 3:** The genomic profiles of SBC detected by NGS have been reported in the literature

Author	year	cases	ETV6- NTRK3 fusion	Other variant gene		prognosis
Krings et al [5]	2017	1	+	*IRS2*		NA
		2	+	-		NA
		3	+	*SOX9*		NA
		4	+	*SMARCA4*		NA
		5	+	-		NA
		6	+	*CHD4*		NA
		7	+	*RASA1*		NA
		8	+	-		NA
		9	+	-		NA
Hoda et al [18]	2019	1primary lesion	+	16p13.3 amplification		12 months Recurrence and metastasis
		1metastasis lesion	+	*TERT CDKN2A*		ANED
		2primary lesion	+	-		45months Recurrence and metastasis
		2metastasis lesion	+	9p21.3 loss		ANED
Maund et al [12]	2022	7 cases	+		*FGF4, FGF3, TERT, EMSY, CDH1, ZNF703, PTEN, PIK3CA, PIK3C2B, MDM4, MCL1*	NA
		2 cases	-	*LMNA ‐ NTRK1*		NA
		2 cases	-	*TPM3 ‐ NTRK1*		NA
Chen et al [2]	2022	2 cases	+	*UGT1A1*		NA

*NA* Not available, *ANED* alive with no evidence of disease

It is generally believed that most SBCs have a triple-negative phenotype or weak positive expression of hormone receptors, and the cases of these two phenotypes have similar signaling pathways [5]. Moderate to strong positive cases of hormone receptors also exist, while cases of HER2 overexpression are extremely rare [4, 15, 16]. However, whether there are differences in the genomic characteristics of such cases has not been reported. In our study, 1 lesion was moderately to strongly positive for ER with approximately 30% neoplastic cells, 2 lesions were weakly positive for ER, and the remaining 4 lesions were triple negative. The rate of moderate to strong positive ER staining among our cases was approximately 20% (1/5), which was similar to that of SBCs reported in the literature (range, 5% to 30%) [4]. Several moderately ER-positive cases in Krings' study had no additional variants except for *ETV6-NTRK3* gene fusion [5]. Two fusion sites, including 5 of *ETV6* and exon 15 of *NTRK3* and exon 6 of *ETV6* and exon 15 of *NTRK3,* were detected in cases with moderate to strong positive staining for ER, and *PGR* mutations were also detected. *PGR* encodes proteins belonging to the steroid receptor superfamily that mediate the physiological effects of progesterone and play a central role in reproductive events related to the establishment and maintenance of pregnancy [31]. Among its related pathways are “mammary gland development pathway - puberty (stage 2 of 4)” and “signal transduction”. A number of studies have reported associations between *PGR* gene variants and hormone-related disorders, including breast cancer, where *PGR* gene mutation more frequently affects ER-positive breast cancer [31, 32].

In one patient, two asynchronous lesions were both weakly positive for ER expression and had different *ETV6-NTRK3* fusion sites, and no other alterations were found. The interval for onset of the two lesions was more than 10 years, and no evidence of other site involvement was found. Because of the different gene fusion sites, the two tumors may be double primary tumors, and their similar morphological characteristics and immunophenotypes may be attributed to the unity of the host environment. Similar to common invasive ductal carcinoma, there can be double primary cases of secretory carcinoma. Only Castillo *et al* reported double primary SBCs in which a patient developed SBC at 14 years old and had a second SBC at 30 years old in the same breast, but the second lesion had a different CGH-array profile [4]. The genomic characteristics of such tumors are not different from those of single tumors but provide clues for the choice of treatment for such tumors.

In this study, the patient who developed distant metastasis and chest wall recurrence had the most aggressive behavior and the most complex genome. The fusion site of *ETV6-NTRK3* was the same as that in distant metastatic cases reported in the literature [18]. Hoda *et al* reported two distant metastases of SBC, one of which had concurrent chest wall recurrent lesions [18]. Sequencing of one chest wall recurrent lesion showed *TERT* C228-T promotor mutation, while the primary lesion in the other showed no *TERT* mutation. The incidence of *TERT* gene promoter mutation in solid tumors is more than 10%, which can generate a new ETS/TCF common binding motif (AAGGCC), promote *TERT* gene transcription, and enhance telomerase activity. ETS transcription factor binding can promote hTERT transcription factors such as C-MYC and SP1 binding to it in a specific microenvironment [33]. Their respective sites upregulate hTERT transcription and telomerase high expression, activate the MAPK signaling pathway, promote tumor transformation, survival, proliferation and invasion, and participate in the occurrence and development of tumors [34]. TCF transcription factor binding can promote the activation of the Wnt/β-catenin signaling pathway, which is involved in the occurrence and development of tumors and affects prognosis [35, 36]. Targeted gene therapy based on the telomerase hTERT promoter provides a new approach for the treatment of tumors [37, 38]. All the evidence suggests that *TERT* promoter mutation may be involved in the progression of SBCs, especially those that undergo distant metastasis. In addition to *TERT* promoter mutation, another mutation is the *STAT3* mutation, which has a very low allele frequency (1%) in recurrent lesions, indicating that this gene may not participate in the tumorigenesis and progression of the disease. Comparing the gene expression profiles of the primary and secondary lesions, the lesions had the same *ETV6-NTRK3* fusion gene, and the primary lesions also had *DOT1L*, *KDM5A* and *LRP1B* variants. Diseases associated with *DOT1L* include NUT midline carcinoma and acute promyelocytic leukemia. Among *DOT1L* related pathways are the PKMT pathway, which involves the methylation of histone lysines, and chromatin organization. Variants of *DOT1L* have been detected in triple-negative breast cancer [39]. *LRP1B*, which encodes endocytic LDL-family receptor, is among the top 10 significantly mutated genes in human cancer [40]. It has been demonstrated that *LRP1B* can bind to multiple extracellular ligands, including fibrinogen and apo E-carrying lipoproteins [40, 41]. Frequent inactivating mutations of LRP1B have been observed in many malignant tumors, including triple-negative breast cancer, whereas its mutation could still have a functional consequence in tumorigenesis and heterogeneity [40, 41]. The *KDM5A*-encoded protein plays a role in gene regulation through the histone code by specifically demethylating lysine 4 of histone H3 [42]. The encoded protein interacts with many other proteins, including retinoblastoma proteins, and is involved in the transcriptional regulation of Hox genes and cytokines [43]. The gene may play a role in tumor progression, which has been detected to be abnormally expressed in triple-negative breast cancer [44]. Variations in all three of these genes may participate in the progression of the tumor. In addition to the *ETV6-NTRK3* gene fusion, *PGR*, *TERT*, *DOT1L*, *KDM5A* and *LRP1B* mutations may be passenger mutations that promote tumor progression. To date, the genomic profiles of only three SBC patients with distant metastases have been reported, including that of one patient in our study. Compared with the SBC genomic profile reported without distant metastases, patients with recurrence or metastasis had more complex genomic alterations [2, 11, 13, 18, 22]. This case had axillary lymph node metastasis at the time of initial diagnosis, and the genome of this case was relatively complex compared to that of the cases with axillary lymph node metastasis that had a favorable prognosis reported in the literature [2].

The treatment regimens in our study included lumpectomy, modified radical mastectomy and modified radical mastectomy with adjuvant radiotherapy, chemotherapy or endocrine therapy. According to the literature, although there are cases of rapid progression, most tumors show an indolent growth process that requires observation and follow-up for more than 10 years. The current study shows that this group of cases lacks a unified treatment regimen, as indicated by clinical trials and literature reports targeting TRKs, which may have therapeutic value in NTRK rearrangement-related malignant tumors, including SBC [45]. The treatment can be done relatively early, rather than waiting for other treatments to work. Current studies suggest that in addition to the fusion of the *ETV6-NTRK3* gene, some advanced cases may be co-driven by other mutated genes, so it is necessary to accumulate more evidence and find more therapeutic targets.

In conclusion, our study demonstrates the genomic characteristics of a group of SBCs with differences in immunophenotype and biological behavior. In addition to *ETV6-NTRK3* gene fusion, *TERT* promoter region mutation may be another factor driving tumor progression. This provides insight into the genomic background and clinical treatment of SBCs.

### Take home messages

The *ETV6-NTRK3* fusion was the main driver of early tumorigenesis in most SBCs.

SBC with invasive biological behavior had more complex genomic variations including *TERT* promoter mutation.

TRKs may have therapeutic value in *NTRK* rearrangement SBCs.

## Data Availability

Data are available upon reasonable request to the authors.
